# Lenvatinib as a Promising Treatment Option for Unresectable Hürthle Cell Carcinoma: A Case Report

**DOI:** 10.7759/cureus.37460

**Published:** 2023-04-11

**Authors:** Parth S Shirode, Anil D'cruz, Sataksi Chatterjee, Swayambhu Bhandarkar

**Affiliations:** 1 Head and Neck Surgery, Apollo Hospitals, Navi Mumbai, IND

**Keywords:** thyroid nodule, lenvatinib, tyrosine kinase inhibitors (tki), hurthle cell carcinoma, thyroid cancer

## Abstract

Hürthle cell carcinoma (HCC) of the thyroid gland generally has a more aggressive clinical course than other differentiated thyroid cancers (DTCs), and it is associated with a higher rate of distant metastases. In this case report, we highlight the importance of tyrosine kinase inhibitors as a management strategy for unresectable DTCs. Surgical management is challenging if the cancer is locally advanced and invades major neck structures with an increased risk of recurrence. Tyrosine kinase inhibitors (TKIs) are used in the case of advanced disease, especially in unresectable, radio-iodine refractory and with metastatic status. Lenvatinib, a TKI, used as the first line of treatment, plays a key role in improving prognosis and survival rates among patients. A 37-year-old gentleman presented with a locally advanced and widely metastasized case of large Hürthle cell carcinoma encasing the left carotid sheath and the left recurrent laryngeal nerve. Fine needle aspiration cytology (FNAC) was suggestive of HCC and a positron emission tomography-computed tomography (PET-CT) scan revealed metastases to the lungs and spine. In this case, lenvatinib was used to prevent the proliferation of malignant cells and the neovascularization of the tumor. This clinically translated into a good response in a high disease burden scenario. The patient showed positive results with lenvatinib therapy with a progression-free duration of 30 months and a reduction in the size of cancer. This case report describes the use of lenvatinib for the treatment of a large unresectable locally advanced and widely metastasized case of Hürthle cell carcinoma in a young gentleman with a response profile.

## Introduction

Hürthle cell carcinoma (HCC) is one of the most aggressive cancers of the thyroid gland, with a tendency to develop distant metastases, which is the ultimate cause of mortality [1]. Although only 3-15% of thyroid cancer patients present with metastasis, around 6-20% may eventually develop metastasis along the course of their disease [2]. Carotid sheath encasement, laryngotracheal invasion, and unilateral recurrent laryngeal nerve invasion, with a high disease burden, make differentiated thyroid cancer (DTC) technically challenging to operate due to the serious consequences of attempting resection. Thyroidectomy is the preferred treatment for operable HCC. Adjuvant therapy can be given after the cancer resection to treat distant metastases. Tyrosine kinase inhibitor (TKI) treatment is reserved for cancer that is unresectable, radio-iodine refractory, and presents with metastatic status [3]. When HCC is locally aggressive and has distantly metastasized, it adversely impacts the quality of life and reduces the overall survival rate. Hence, a physician must be well aware of its complications and various treatment modalities. This case report describes the use of lenvatinib, a TKI, for the treatment of a large unresectable locally advanced, and widely metastasized case of Hürthle cell carcinoma in a young male with a response profile.

## Case presentation

**Figure 1:**
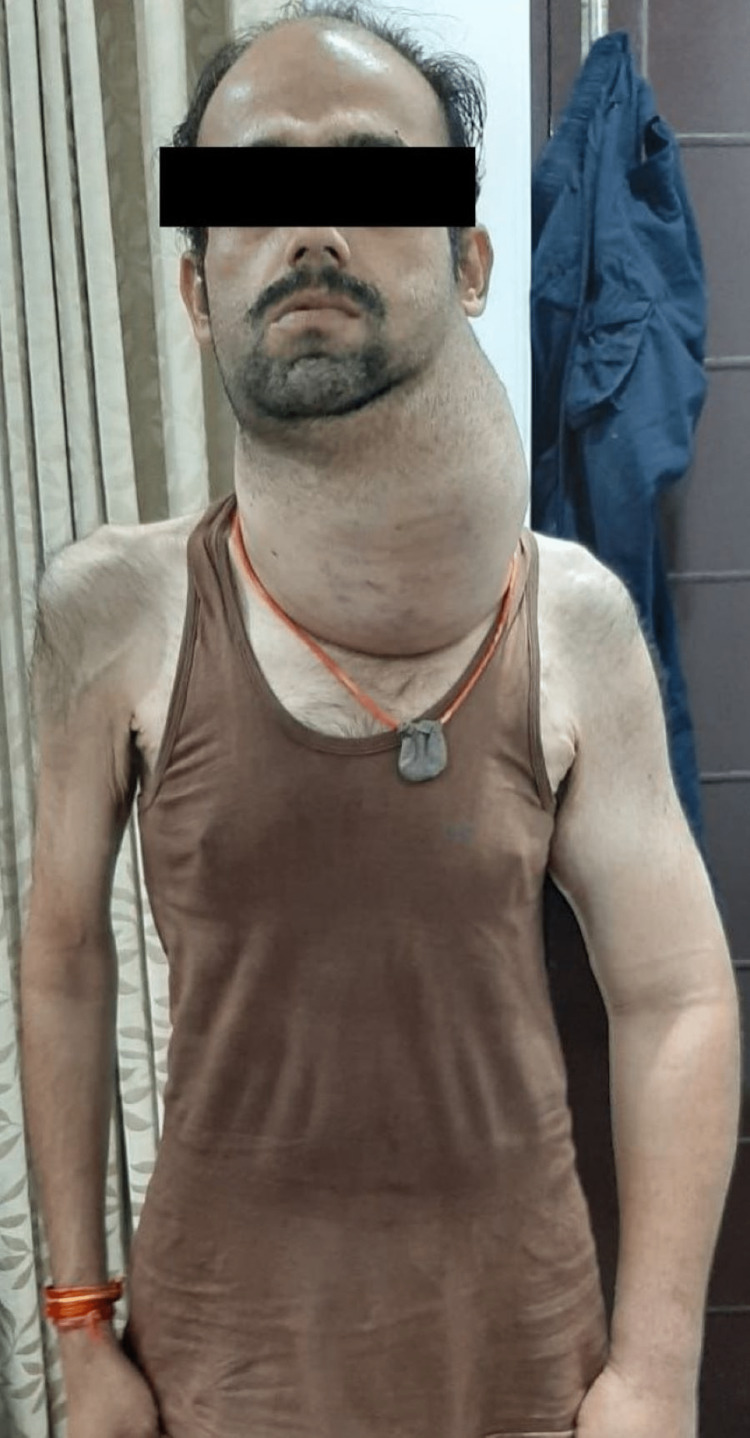
The patient with Hürthle cell carcinoma before lenvatinib therapy 11.7 cm x 14.1 cm x 17.7 cm sized lobulated soft tissue mass on the left side of the neck and generalized swelling due to lymphoedema of the left upper extremity.

A 37-year-old gentleman presented with a large swelling on his neck which first appeared 16 years back. It was gradually increasing in size over 13 years for which no treatment was sought by the patient. Three years back, he developed rapidly increased swelling, weight loss, generalized swelling due to lymphoedema of the left upper extremity, and hoarseness of voice (Figure 1). A diagnosis of Hürthle cell carcinoma of the thyroid gland was made based on fine needle aspiration cytology (FNAC) with very high (300,000 ng/ml) levels of serum thyroglobulin (Sr. TG). He was also detected to have subclinical hypothyroidism with low levels (0.16ug/dL) of serum free tetra-iodo-thyroxine (Sr. FT4) and normal levels of serum thyroid stimulating hormone (Sr. TSH) and free tri-iodo-thyroxine (Sr. FT3).

**Figure 2:**
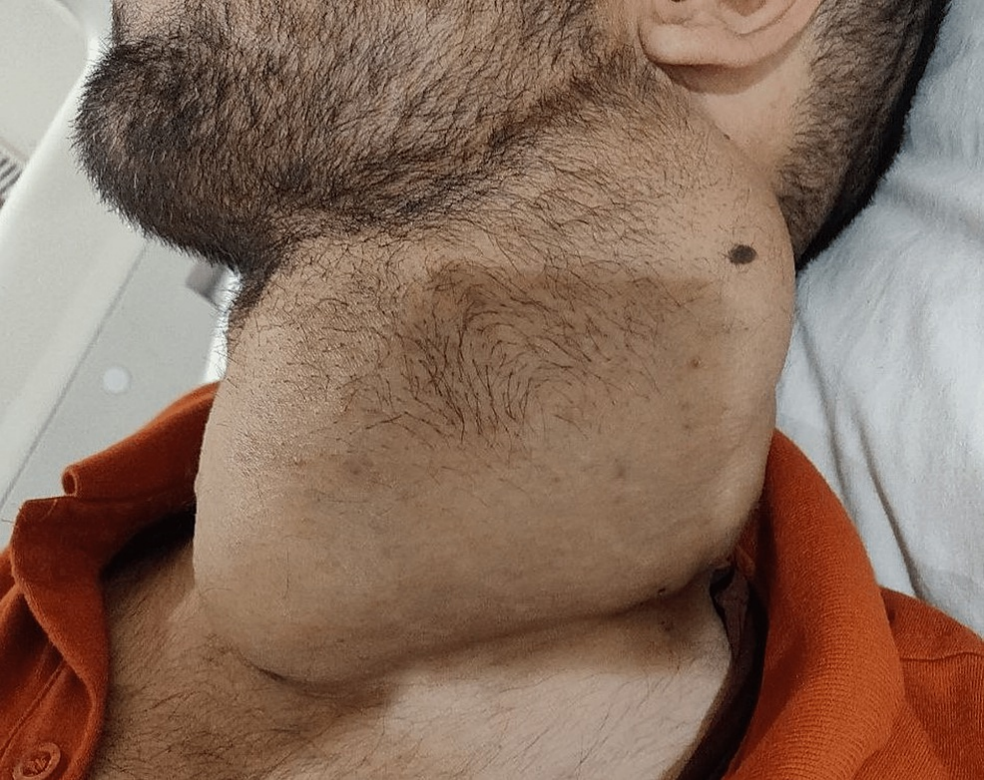
Reduced tumor size after 30 months of lenvatinib therapy - 9 cm x 8.7 cm x 13.5 cm

A magnetic resonance imaging (MRI) revealed an extremely large, 11.7 cm x 14.1 cm x 17.7 cm sized lobulated soft tissue mass on the left side of the neck extending superiorly from submandibular-submental space to inferior into the mediastinum upto the carina, lateral deviation of the common carotid artery with partial encasement of the internal and external carotid artery and severe compression of internal jugular vein by soft tissue. Magnetic resonance imaging (MRI) also revealed conglomerated nodal mass along the left side of the neck at II, III, IV, Va, and Vb levels, with some showing cystic changes. Due to the unresectability of cancer, the patient was started on oral lenvatinib 20 mg once daily. The patient responded to the treatment over 2.5 years (30 months) with a significant decrease in the size of swelling over the neck, complete recovery of left upper extremity lymphoedema, weight gain, and overall improvement in well-being (Figure 2).

Six months back, he presented with loss of appetite, constipation, asthenia, and overall deteriorating health. Investigations revealed a reduction in levels of Sr. FT4 to 0.43 µg/dl, and an increase in levels of Sr. TSH to 150 µIU/ml; Sr. FT3 and Sr. thyroglobulin levels were normal (2.67 pg/mL and 540 ng/ml respectively) suggestive of hypothyroidism. Due to worsening hypothyroidism, the patient was started on oral thyroxine 100 mcg once daily with a dose escalation of lenvatinib to 24 mg once daily. Consequently, Sr. TSH levels dropped to 22 µIU/ml and Sr. FT4 levels returned to normal after 4 months.

**Figure 3:**
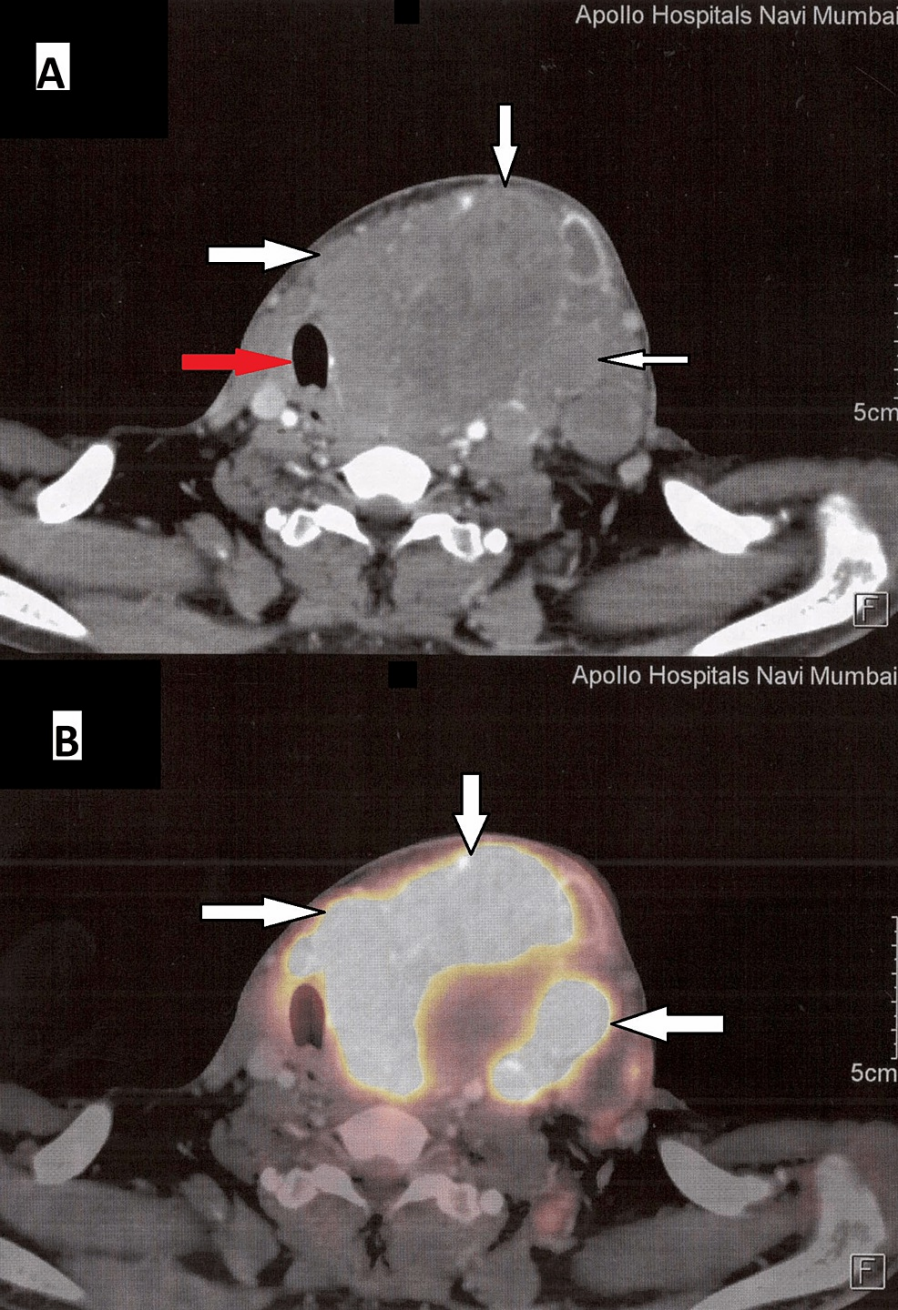
Positron Emission Tomography-Computed Tomography (PET-CT) scan showing soft tissue swelling around the neck. A) Computed Tomography scan showing soft tissue density around the neck involving the left lobe of the thyroid gland (white arrow). The mass compresses and displaces the trachea to the contralateral side (Red arrow). B) PET-CT scan showing enhancing soft tissue mass (white arrow) involving the left lobe of the thyroid gland with increased fluorodeoxyglucose (FDG) uptake (Max SUV:77.11). It also infiltrates the isthmus and the right lobe.

**Figure 4:**
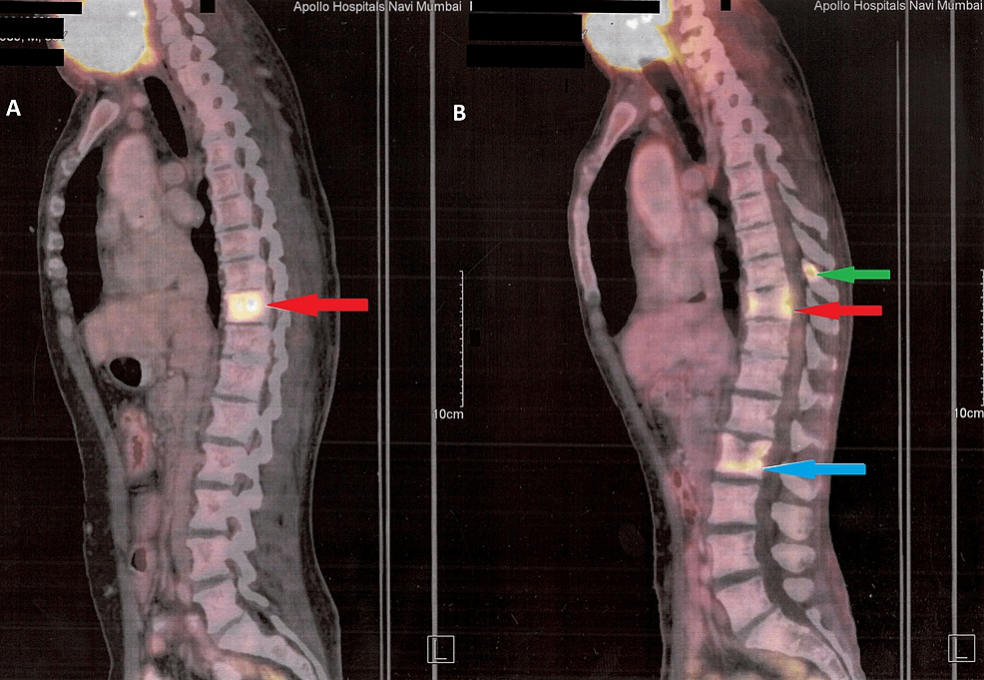
Positron Emission Tomography-Computed Tomography (PET-CT) scan showing metastasis to vertebrae A) lncreased fluorodeoxyglucose (FDG) uptake in the marrow and lytic lesion involving thoracic D10 vertebrae (red arrow) B) lncreased FDG uptake in the marrow and lytic lesions involving thoracic D9 vertebrae (green arrow), D10 vertebrae (red arrow), and lumbar L2 vertebrae (blue arrow)

Current positive emission tomography-computed tomography (PET-CT) scan revealed an active disease with enhancing soft tissue swelling of size 9 cm x 8.7 cm x 13.5 cm involving the left lobe of the thyroid (Figure 3), metastatic left cervical level II to V nodes, left retropharyngeal and mediastinal nodes, a metastatic nodule in the right lower lobe of the lung, left mediastinal pleural deposits, marrow metastasis and lytic lesions involving thoracic D9-D10 and lumbar L2 vertebrae (Figure 4) and left inferior pubic ramus. In the past four months, he has also developed back pain. Palliative radiations of 40 Gray in 10 cycles were given to these areas, and oral morphine 10 mg once daily to relieve back pain. The patient was advised to have a monthly follow-up.

Throughout the course of treatment, his weight grew from 45 kgs to 60 kgs, his appetite increased, his left upper limb lymphedema was cured totally, Sr. Thyroglobulin levels were lowered from 300,000 ng/ml to 540 ng/ml, and the most critical, the tumor size decreased from 11.7 cm x 14.1 cm x 17.7 cm to 9 cm x 8.7 cm x 13.5 cm. Over the course of 30 months, Eastern Cooperative Oncology Group - Performance Status Scale (ECOG-PS) grade 3 improved to grade 1.

## Discussion

Thyroid carcinomas fall into three categories: Differentiated (Hürthle cell, follicular, and papillary), poorly differentiated, and undifferentiated (anaplastic) carcinoma. The occurrence of thyroid cancer in the age group of 20-34 is 16.2%, with an incidence in males being 7.7 per 100,000 persons. The proportion of women to men is 3.6:1 [4]. Fewer than three to five percent of differentiated thyroid cancers are Hürthle cell carcinomas (HCC). Hürthle cell carcinomas were formerly considered a variety of follicular thyroid carcinomas; however, they were designated as a separate entity in the 2017 World Health Organization (WHO) classification due to biological behaviors and molecular profiles that differed from follicular tumors [5]. The differentiated subtype of thyroid cancer known as follicular thyroid carcinoma has a higher propensity for distant metastasis than papillary thyroid carcinoma. However, HCC exhibits an even larger predisposition for distant metastasis to organs like the lungs and bone, due to the propensity for vascular invasion [6]. Besic et al. in a retrospective investigation of 32 patients with metastatic HCC reported estimated 5-year and 10-year disease-specific survival rates of 70% and 38%, respectively, from the detection of distant metastases [7].

Two factors play a key role in the management of this cancer. First, the size of cancer, and second, the extent of local advancement of cancer. The treatment protocol for thyroid cancers of any size invading local structures is total thyroidectomy with extended resection [8]. Radioiodine therapy with thyroxine and systemic chemotherapy is recommended as adjuvant therapy in advanced metastatic cancers with a high risk of recurrence after cancer resection [9]. Early thyroid stimulating hormone (TSH) suppression with levothyroxine therapy to less than 0.1 µIU/ml is advised for thyroid cancer patients at high risk [10]. If the tumor is large and encroaching on the major nerves and vessels of the neck region, it is considered unresectable due to the grave consequences of attempting resection. Hence measures should be taken to reduce the size of a tumor to make it operable. 

Tyrosine kinase inhibitors are used in the case of advanced disease, especially in unresectable, radio-iodine refractory differentiated thyroid cancer (DTC), and DTC with metastatic status [3]. HCC has less avidity for radioiodine compared to follicular cell carcinoma due to the loss of thyroid differentiation features, such as iodide uptake and organification. According to estimates, only 10% of individuals with HCC lesions absorb radioiodine; as a result, responses of these patients to radioiodine therapy are significantly lower than those of patients with other forms of thyroid carcinomas [11]. Tyrosine kinase inhibitor (TKI) lenvatinib significantly increases response rate and progression-free survival when compared to placebo [12]. Lenvatinib prevents angiogenesis and further proliferation of malignant cells which eventually suppress cancer growth. The median progression-free survival is 18.3 months and the progression-free survival rate for 24 months is 44.3% [13].

In our case, the patient with a high disease burden responded well to the given line of treatment with a progress-free duration of 30 months, evidenced by the reduction in the size of the cancer from 11.7 cm x 14.1 cm x 17.7 cm to 9 cm x 8.7 cm x 13.5 cm after initiation of lenvatinib therapy. Throughout the course of treatment, our patient's weight increased from 45 kg to 60 kg, his appetite increased, his left upper limb lymphedema was completely cured, Sr. thyroglobulin levels reduced from 300,000 to 540 ng/ml. ECOG-PS grade 3 improved to grade 1 during the course of 30 months. This therapy could not prevent the distant metastasis of cancer cells though it delayed the disease progression. External Beam Radiation Therapy (EBRT) has a crucial role in treating isolated bone lesions as it reduces the risk of pathological fractures. During the initial follow-up of the patient, serum thyroglobulin, thyroglobulin antibody, and TSH levels should be measured every month.

The novel aspect of this case is that it describes the potential and promising role of lenvatinib in a patient with locally advanced, unresectable, radio-iodine refractory thyroid tumor in reducing tumor mass to make it resectable and also providing the patient with longer progression-free survival.

## Conclusions

Hürthle cell carcinoma (HCC) contains more invasive and metastatic potential than any other differentiated carcinomas of the thyroid gland. Lenvatinib, a tyrosine kinase Inhibitor, is effective in treating unresectable HCC and can significantly increase progression-free survival. In our case, the patient showed positive results with lenvatinib therapy with a progression-free duration of 30 months, a significant reduction in the size of cancer, and an overall improvement in well-being.
